# Dinutuximab Beta in Children with High-Risk Neuroblastoma: Experience from a Single Center in Croatia

**DOI:** 10.3390/children9070943

**Published:** 2022-06-23

**Authors:** Jasminka Stepan Giljević, Nada Rajačić, Danko Mikulić, Ana Tripalo Batoš

**Affiliations:** 1 Department of Pediatric Oncology and Haematology, Children’s Hospital Zagreb, Ul. Vjekoslava Klaića 16, 10000 Zagreb, Croatia; nada.rajacic@gmail.com (N.R.); abatosh@gmail.com (A.T.B.); 2 Department of Surgery, University Hospital Merkur, 10000 Zagreb, Croatia; mikulicdanko@gmail.com

**Keywords:** neuroblastoma, dinutuximab beta, immunotherapy, adverse events, outcomes, maintenance

## Abstract

To determine the potential benefits and feasibility of administering maintenance therapy with dinutuximab beta for high-risk neuroblastoma (HRNB) in clinical practice, a retrospective review of charts of patients with HRNB treated at a single center in Croatia (2012–2021) was undertaken. Of 23 patients with HRNB, 11 received up to five cycles of dinutuximab beta as part of multimodal therapy; 12 patients did not (i.e., no immunotherapy). In the no immunotherapy group, one patient had complete remission (8%), and 11 patients died of tumor progression (92%). In the dinutuximab beta group, eight patients had complete remission (73%; median duration of response 5 years and 2 months), one had stable disease (9%), and two died of disease (18%). Patients who received dinutuximab beta had a higher median event-free survival (40.0 months [range: 12.5–83.0]) and median overall survival (56.0 months [range: 16.2–101.0]) than those who did not (12.9 months [range: 3.3–126.0] and 20.7 months [3.3–126.0], respectively). Dinutuximab beta was generally well tolerated; adverse events were manageable and as reported in clinical studies. These results confirm the benefits and feasibility of maintenance therapy with dinutuximab beta as part of multimodal therapy for patients with HRNB in real-world clinical practice.

## 1. Introduction

Neuroblastoma is the most commonly diagnosed cancer in infancy (i.e., during the first year of life) and the most common extracranial solid tumor in childhood [1]. It is a tumor of the peripheral sympathetic nervous system that generally affects the abdomen, with most tumors originating in a suprarenal (also known as adrenal) gland [1].

Worldwide, the incidence of neuroblastoma is estimated between 3 and 15 cases per million children (0–14 years of age) [2], with rates as high as 53 cases per million reported in infants in Europe between 1988 and 1997, during which time incidence rates were increasing by approximately 1.5% annually [3]. Indeed, approximately one-third of all cases of neuroblastoma are diagnosed during infancy [2]. In Croatia, the age-adjusted incidence of neuroblastoma was 13.2 cases per million children (0–14 years of age) between 2000 and 2014, with a crude incidence of 51.6 cases per million in infants [2].

Neuroblastoma is a highly heterogeneous disease that can have a benign course or result in a terminal illness [1]. Due to this heterogeneity, the treatment of neuroblastoma is based on each patient’s risk of relapse and death, which is stratified into low, intermediate, and high using specific criteria alone or in combination, including age, *MYCN* oncogene amplification, metastatic disease, and histology/pathology [4]. While survival is almost 100% in patients with low- or intermediate-risk neuroblastoma, the five-year survival rate in patients with high-risk neuroblastoma (HRNB) is less than 50% [5].

The introduction of intense multimodal therapy for patients with HRNB, which includes induction chemotherapy, surgery, consolidation myeloablative chemotherapy (MAT) followed by autologous stem cell transplantation (ASCT), radiation therapy, and maintenance immunotherapy, significantly improved outcomes in these patients [6,7,8,9,10,11]. In the HR-NBL1 study, a clinical trial led by the International Society of Paediatric Oncology European Neuroblastoma (SIOPEN) group, the use of maintenance therapy with the anti-GD2 antibody dinutuximab beta as part of multimodal treatment for patients with HRNB increased five-year overall survival (OS) to over 60% [8].

Based on the positive results of the SIOPEN trials [8,12,13], dinutuximab beta was approved in Europe in May 2017 for the treatment of HRNB in patients ≥12 months of age who have achieved at least a partial response to induction chemotherapy, followed by MAT and ASCT, and in patients with a history of relapsed or refractory neuroblastoma with or without residual disease [14]. Five cycles of dinutuximab beta should be administered either as continuous infusion for the first 10 days of each 35-day cycle (10 mg/m^2^ per day; cumulative dose 100 mg/m^2^) or as short 8-h infusion for the first 5 days of the cycle (20 mg/m^2^ per day; cumulative dose 100 mg/m^2^) [14]. According to the prescribing information, dinutuximab beta should be administered in combination with interleukin-2 (IL-2) in patients with a history of relapsed/refractory disease and in those who did not achieve a complete response after first-line therapy [14].

The SIOPEN group recommends dinutuximab beta alongside isotretinoin as the standard of care for patients with HRNB [9,12,15]. Based on the results of the HR-NBL1 study, SIOPEN concluded that administering dinutuximab beta via a continuous infusion rather than a short 8-h infusion improves its tolerability, as does administering it without IL-2 [12]. The German Pediatric Oncology and Hematology Group (Gesellschaft für Pädiatrische Onkologie und Hämatologie; GPOH) guidelines also recommend treating patients with HRNB with dinutuximab beta and without IL-2 [16].

Currently, there is limited published information regarding the use of dinutuximab beta in real-world clinical practice. Data from real-world settings complement those from clinical trials, indicating treatment effectiveness in routine clinical practice and providing data in a broader range of patients than those typically included in clinical trials [17].

Here, we present data from patients with HRNB who received dinutuximab beta maintenance therapy in everyday clinical practice at a single center in Croatia, focusing on outcomes and adverse events (AEs). For context, data are also provided for patients with HRNB treated at the same center prior to the availability of dinutuximab beta (May 2017).

## 2. Methods

A retrospective review of the clinical charts of all patients with HRNB treated at the Children’s Hospital, Zagreb, Croatia between 2012 and 2021 was undertaken, following approval from the local ethics board.

## 3. Results

### 3.1. Patients

In total, 23 patients received treatment for HRNB at the Children’s Hospital, Zagreb, Croatia between 2012 and 2021, including 11 patients who received maintenance therapy with up to five cycles of dinutuximab beta (Table 1, Table 2 and Table 3). The 12 patients who did not receive immunotherapy with dinutuximab beta (i.e., the ‘no immunotherapy’ group) were treated before May 2017 when dinutuximab beta became available.

Patients were classified as having HRNB based on the International Neuroblastoma Staging System (INSS) classification system [4,18] or based on their clinical condition. Patients had HRNB if they were at least 12 months of age with INSS stage 4 neuroblastoma (primary tumor with dissemination to the distant lymph nodes, bone, bone marrow, liver, skin and/or other organs, except as defined for 4S), or <12 months of age with INSS stage 4 disease and *MYCN* amplification [4,18]. In the dinutuximab beta group, patient 3 was treated as a high-risk patient as they had bone lesions that were still evident following induction chemotherapy. Patient 5C, who did not receive dinutuximab beta was considered high risk due to their progressive tumor requiring chemotherapy to be administered in the intensive care unit (ICU) while the patient was receiving mechanical ventilation.

The majority of patients with HRNB were female (15/23; 65%; Table 1), and one in five patients (26%) were diagnosed with neuroblastoma during infancy. The majority of primary tumors were located in a suprarenal gland, with metastases in the bone, bone marrow, lymph nodes, and liver.

### 3.2. Treatment

All patients received intensive induction chemotherapy (Table 2 and Table 3). All 12 patients in the no immunotherapy group were treated with NB2004 (two cycles of N8 [topotecan, cyclophosphamide, etoposide] followed by six alternating courses of N5 [vindesine, cisplatin, etoposide] and N6 [vincristine, dacarbazine, ifosfamide, doxorubicin]) [19], as later recommended by the GPOH [20]. Most patients (73%) who received dinutuximab beta (dinutuximab beta group) also received NB2004. However, following a visit from SIOPEN representatives in June 2018, the standard intensive induction chemotherapy protocol was changed from NB2004 to rapid COJEC (time-intensive cisplatin, carboplatin, cyclophosphamide, vincristine, etoposide), as recommended by SIOPEN, based on the results of the SIOPEN HR-NBL1 study [7,8], and by the European Standard Clinical Practice (ESCP) guidelines [15]. As a result, three patients in the dinutuximab beta group received rapid COJEC induction therapy. One patient in the dinutuximab beta group also received N8 induction therapy (topotecan, cyclophosphamide, etoposide) following a relapse, after initially receiving NB2004.

Most patients underwent tumor resection with or without lymphadenectomy. Surgery was not indicated in one patient in the no immunotherapy group (patient had progressive disease after induction therapy and received chemotherapy in the ICU whilst receiving mechanical ventilation), and in two patients in the dinutuximab beta group due to total regression of the tumor following induction chemotherapy (Table 2 and Table 3).

One patient in the no immunotherapy group and the three patients in the dinutuximab beta group who received rapid COJEC underwent radiotherapy. In the no immunotherapy group, eight patients received therapeutic iodine-131-metaiodobenzylguanidine (^131^I-MIBG; 12 mCi/kg body weight infused over 2 h), as did six of the eight patients who received NB2004 in the dinutuximab beta group. ^131^I-MIBG therapy was given to all patients who demonstrated residual MIBG uptake, in line with the NB2004 protocol. Most patients received MAT (87%), with either busulfan and melphalan (BuMel) and/or carboplatin, etoposide, and melphalan (CEM), followed by a peripheral blood ASCT (Table 2 and Table 3).

All 11 patients in the dinutuximab beta group received up to five cycles of dinutuximab beta maintenance therapy as continuous infusion over 10 days (10 mg/m^2^ per day); one patient relapsed after receiving only one cycle and another stopped after four cycles due to an AE. One patient developed acute myeloid leukemia after completion of immunotherapy and underwent bone marrow transplantation. Concomitant isotretinoin was received by four patients (36%), and concomitant IL-2 by three patients (27%) (Table 3). All patients in the no immunotherapy group received maintenance therapy with isotretinoin alone.

### 3.3. Outcomes

In the no immunotherapy group, one of 12 patients had complete remission (8%; patient 1; follow-up nine years and two months), and the other 11 patients (92%) died due to tumor progression between four months and six years and four months after diagnosis (Table 2). In patients in the dinutuximab beta group, complete remission was achieved in eight of the 11 patients (73%; median duration of remission five years and two months [range one year and three months to six years and two months]), one patient who received four cycles of dinutuximab beta had stable disease, and two patients died due to disease both 1 year and 10 months after diagnosis (one after receiving a single cycle of dinutuximab beta; Table 3). All patients in the dinutuximab beta group had achieved complete remission following MAT and ASCT. Patients with HRNB who received dinutuximab beta maintenance therapy in addition to multimodal therapy had a higher median event-free survival (EFS) (40.0 months [range 12.5−83.0]) and median OS (56.0 months [range 16.2−101.0]) than those who did not (12.9 months [range 3.3−126.0] and 20.7 months [range 3.3−126.0], for median EFS and OS respectively).

### 3.4. Adverse Events with Dinutuximab Beta

Dinutuximab beta was generally well tolerated, and all AEs were manageable. All patients who received dinutuximab beta had an AE during cycle 1, with some patients also having an AE during cycle 2. One patient, who had hypertension during cycle 1, also had renal impairment during cycle 3, and facial paresis during cycle 4 (patient 10), resulting in only four cycles of dinutuximab beta being administered. 

The most common AEs associated with dinutuximab beta in this case series (Table 4) were fever, fluid retention indicative of capillary leak syndrome, hypotension, hypoxia and diarrhea. Hypoxia, which was reported in four patients, was treated with oxygen supplementation. Patients with fluid retention received furosemide.

Two patients reported moderate pain in cycle 1, one of whom also received IL-2. All patients received gabapentin, non-steroidal anti-inflammatory drugs (NSAIDs), paracetamol and morphine prior to and/or during dinutuximab beta treatment to prevent and/or manage pain as recommended [14]. Oral gabapentin was given for 3 days prior to dinutuximab beta infusion (day 1: 1 × 10 mg/kg/day; day 2: 2 × 10 mg/kg/day; day 3: 3 × 10 mg/kg/day) and morphine infusion (0.03 mg/kg/h) was started 2 h before immunotherapy. Paracetamol, ketoprofen, ibuprofen and indometacin were given during immunotherapy.

In addition to the AEs experienced by patient 10 (hypertension, renal impairment, and facial paresis), other AEs experienced by a single patient were tachycardia in cycle 1 (patient 1) and reversible blurred vision in cycle 1 (patient 4). One patient (patient 5) developed acute myeloid leukemia after the completion of dinutuximab beta and underwent bone marrow transplantation; the patient is now in remission.

### 3.5. Single Case Presentation

**Figure 1:**
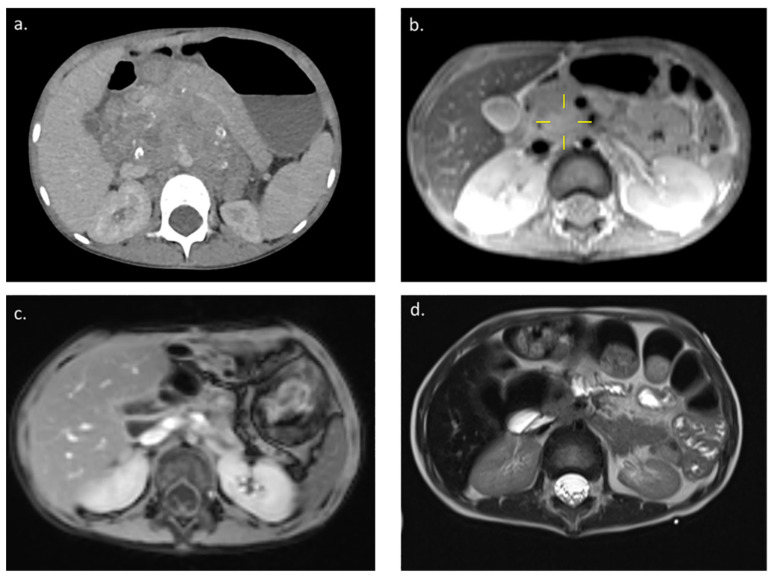
Contrast-enhanced computed tomography image of the abdomen of a girl, three years and six months of age, with high-risk neuroblastoma (**a**) at diagnosis, (**b**) prior to surgery (following rapid COJEC induction chemotherapy), (**c**) prior to receiving dinutuximab beta (after surgery, myeloablative chemotherapy, autologous stem cell transplant, and radiotherapy), and (**d**) after receiving dinutuximab beta. COJEC, cisplatin (C), vincristine (O), carboplatin (J), etoposide (E), and cyclophosphamide (C).

A girl, three years and six months old (patient 9), was admitted to hospital with an extensive intra-abdominal tumor originating from the right suprarenal gland three months after initially presenting with abdominal pain, vomiting, diarrhea, and significant weight loss. Abdominal computed tomography revealed a retroperitoneal tumor (craniocaudal diameter [CC] 11 cm, anteroposterior [AP] diameter 5.0 cm, laterolateral [LL] diameter 8.8 cm) that spread from the subhepatic area to the bifurcation of the aorta to the iliac blood vessels (Figure 1a). Mesenteric blood vessels were involved, dislocated hepatic and splenic artery and elongated celiac trunk, with ventral shift of the pancreas. Poorly differentiated neuroblastoma without *MYCN* amplification was diagnosed from a biopsy specimen. However, as demonstrated by bone marrow aspiration cytology, tumor cells were invading the bone marrow and Technetium scintigraph identified multiple sites of bone metastases.

The patient was treated according to the SIOPEN HR-NBL1 protocol [7,8]. Stem cells were collected following rapid COJEC induction therapy. Prior to undergoing surgery, magnetic resonance imaging (MRI) indicated a good response to induction chemotherapy with tumor shrinkage (CC 4.5 cm, AP 2.0 cm, LL 3.1 cm) (Figure 1b). While, the tumor still involved the celiac trunk, the mesenteric blood vessels were no longer involved. The patient underwent surgery, during which the tumor was found to be firmly attached to the head of the pancreas and fully incorporated the arteria mesenterica inferior and the celiac trunk. A radical maximal resection of the tumor and Whipple procedure were performed (pancreaticoduodenectomy with pancreaticojejunostomy, hepaticojejunostomy, and gastrojejunostomy with cholecystectomy). The patient then received consolidative MAT with BuMel followed by ASCT. Her recovery was slow due to diarrhea and the need for parenteral nutrition. Following radiotherapy for the abdomen, there was no tumor recurrence on MRI (Figure 1c) and the bone marrow was clear of tumor cells. The patient’s catecholamine levels and neuron-specific enolase levels were within the normal range.

The patient was started on dinutuximab beta and received five cycles in total. She experienced febrile episodes, fluid retention, and diarrhea during treatment and was managed using antihistamines, NSAIDs, and furosemide, with occasional oxygen supplementation. There was no evidence of disease following immunotherapy (Figure 1d). As of May 2022, the patient, 6 years and 11 months of age at that time, had no signs of residual disease and no late treatment complication.

## 4. Discussion

Despite therapeutic advances, HRNB has a poor prognosis. Clinical trials led by the SIOPEN group have shown that immunotherapy with dinutuximab beta as part of multimodal treatment improves survival in patients with HRNB [8,9,12,13]. However, published literature on the use of this therapy in real-world settings is limited. Here we demonstrate the feasibility and clinical benefits of dinutuximab beta in 11 patients with HRNB who have been treated under clinical practice conditions at our center in Zagreb. For context, we also report the outcomes of another 12 patients who were diagnosed with HRNB at our center but were treated before dinutuximab beta became available.

In line with the standard of care, all our patients were treated with multimodality treatment, starting with intensive induction chemotherapy. While the majority of patients received NB2004 therapy as recommended by the GPOH, three were treated with rapid COJEC. The efficacy of rapid COJEC and GPOH N5/N6 induction regimens are being compared in the HR-NBL2 study, into which participants are currently being recruited (clinicaltrials.gov NCT04221035). Most of our patients underwent surgery and received ^131^I-MIBG and consolidation therapy with BuMel and CEM followed by ASCT. In 2017, SIOPEN recommended BuMel as the standard of care in Europe as it improved EFS and was better tolerated than CEM in patients with HRNB in the HR-NBL1 study [7]. Consolidation therapy with BuMel is also recommended in the ESCP guidelines [15].

All patients in the no immunotherapy group received isotretinoin as maintenance therapy, whereas patients in the dinutuximab beta group were treated with either dinutuximab beta alone or in combination with isotretinoin or IL-2. In the HR-NBL1 SIOPEN study, patients received dinutuximab beta and isotretinoin with or without IL-2 [8]. While the addition of IL-2 to dinutuximab beta did not improve EFS and OS rates compared with dinutuximab beta alone (five-year EFS: 57% versus 53%; five-year OS: 62% versus 63%), it was associated with increased toxicity [8]. The patients who received IL-2 in our study were treated before these findings were published.

A historical control analysis by the SIOPEN group compared the survival outcomes with dinutuximab beta in the NR-NBL1 trial with those of a historical control group who were treated with the previous standard of care, i.e., isotretinoin [9]. The findings demonstrated that dinutuximab beta ± IL-2 added to isotretinoin significantly improved EFS and OS versus isotretinoin alone (five-year EFS: 57% versus 42%; five-year OS: 64% versus 50%; *p* < 0.001). In our analysis of real-world data, patients who received dinutuximab beta also had a higher median EFS (40.0 months) and OS (56.0 months) than those who did not receive it (12.9 months and 20.7 months, respectively). The majority of patients (73%; 8/11) in the dinutuximab beta group achieved complete remission, with a median duration of five years and two months. Of the 12 patients who did not receive immunotherapy, only one patient had complete remission. However, there was an imbalance between the two groups with regard to response prior to maintenance treatment which might have contributed to the positive outcomes in patients treated with dinutuximab beta. In a multivariate analysis carried out by the SIOPEN group, less than complete remission prior to maintenance therapy, type of MAT and absence of immunotherapy were identified as risk factors for relapse or progression [9]. Response prior to immunotherapy was found to be an important prognostic factor: patients in complete remission treated with immunotherapy had a two-year EFS rate of 68% compared with 54% in the historical control group [9].

Dinutuximab beta was generally well tolerated in our patients, with fever, fluid retention, hypotension, hypoxia and diarrhea being the most frequently reported AEs. In line with data from the HR-NBL1 SIOPEN study, the tolerability profile of dinutuximab beta generally improved with subsequent cycles [8], and all AEs were manageable with supportive therapy. Pain, which was one of the most common AEs in patients receiving dinutuximab beta in clinical trials [8,21], was well managed with analgesics as recommended [14]. Only two patients reported moderate pain in the current case series, one of whom also received IL-2, which has been shown to be associated with a higher incidence of pain when combined with dinutuximab beta [8,21]. The feasibility, tolerability and effectiveness of dinutuximab beta administration in real-world clinical practice was also demonstrated in a recent single-center experience in Slovakia [22,23]. In this small case series, dinutuximab beta resulted in complete remission in six of seven patients with HRNB, and was tolerable in most patients, with the majority of AEs managed with supportive therapy [23].

Despite several study limitations, including the small sample size, the retrospective nature and the short follow-up, the findings of our study add to the available real-world evidence of dinutuximab beta treatment in patients with HRNB.

## 5. Conclusions

These results confirm that the benefits of maintenance therapy with dinutuximab beta in patients with HRNB reported in clinical trials are also seen in real-world practice, and demonstrate the feasibility of including dinutuximab beta in the multimodal treatment of these patients in routine clinical practice.

## Figures and Tables

**Table 1:** Summary of disease characteristics for all 24 patients with high-risk neuroblastoma treated with or without immunotherapy.

Characteristic	No Immunotherapy (n = 12)	Dinutuximab Beta (n = 11)
**Age at diagnosis, median (range)**	2 yrs	2 yrs 2 m
**Sex**		
**Female**	8	7
**Male**	4	4
**Mutations**		
***MYCN* amplified**	7	5
**Del (1p) positive**	1	1
**INSS [18]**		
**Stage 4**	12	11
**Histology**		
**Poorly differentiated**	NA ^a^	6
**Not otherwise specified**	NA ^a^	5
**Primary tumor ^b^**		
**Suprarenal gland**	8	7
**Retroperitoneum**	4	1
**Mediastinum**	2	1
**Intra-abdominal**	0	2
**Metastases**		
**Bone marrow**	8	6
**Bone**	3	9
**Lymph nodes**	5	1
**Liver**	2	2
**Lung**	0	1
**Pancreas**	0	1

^a^ All patients in the no immunotherapy group had tumors that were poorly differentiated or not otherwise specified, based on the Shimada classification; however, the exact numbers are unavailable. ^b^ Two patients in the no immunotherapy group had a primary tumor in more than one location. Del, deletion; INSS, International Neuroblastoma Staging System; m, months; NA, not available; yrs, years.

**Table 2:** Disease characteristics, treatment and outcomes for 12 patients with high-risk neuroblastoma who did not receive immunotherapy.

Pt	Sex	Age at Diagnosis	INSSStage [18]	Primary Tumor (Pathophysiologic Findings) ^a^	Metastases	Mutations *MYCN* del(1p)		Treatment and Status of Disease						Outcome (Last FU)
							Induction Chemotherapy	Status after Induction Chemotherapy	Surgery	RT	^131^I-MIBG	MAT	Status after MAT and ASCT	
1C	M	6 yrs 8 m	4	Right suprarenal gland	BM	*MYCN* not amplified del(1p) negative	NB2004	VGPR	√	–	√	CEM	CR	Complete remission duration: 9 yrs 2 m (last FU October 2021; 15 yrs 9 m of age)
2C	F	2 yrs 4 m	4	Right suprarenal gland	BM	*MYCN* not amplified del(1p) negative	NB2004	PR	√	–	√	CEM	VGPR	Died of disease: tumor progression 5 yrs 9 m of age
3C	M	1 yr 6 m	4	Right suprarenal gland	BM, bone	*MYCN* not amplified del(1p) negative	NB2004	MR	√	–	√	CEM	VGPR	Died of disease: tumor progression 3 yrs 8 m of age
4C	F	10 yrs 1 m	4	Right mediastinum	Lymph nodes—neck	*MYCN* not amplified del(1p) negative	NB2004	MR	√ ^b^	–	√	CEM	PR	Died of disease: tumor progression 12 yrs 8 m of age
5C	F	3 m	4	Right suprarenal gland	Liver, intra-abdominal lymph nodes	*MYCN* not amplified del(1p) negative	NB2004	PD	–	–	–	–	–	Died of disease: tumor progression 7 m of age
6C	F	1 yr 7 m	4	Left suprarenal gland	Intra-abdominal and neck lymph nodes, BM	*MYCN* amplified del(1p) negative	NB2004	VGPR	√ ^b^	–	√	CEM	VGPR	Died of disease: tumor progression 4 yrs 11 m of age
7C	F	1 yr 8 m	4	Retroperitoneum, left suprarenal gland	Intra-abdominal lymph nodes	*MYCN* amplified del(1p) negative	NB2004	PR	√ ^b^	–	√	CEM	–	Died of disease: tumor progression 2 yrs 1 m of age
8C	F	7 yrs 7 m	4	Left suprarenal gland	BM	*MYCN* amplified del(1p) negative	NB2004	MR	√	–	√	CEM	VGPR	Died of disease: tumor progression 8 yrs 11 m of age
9C	M	1 yr 4 m	4	Retroperitoneum, mediastinum	BM, intrathoracic and intra-abdominal lymph nodes	*MYCN* amplified del(1p) positive	NB2004	VGPR	√ ^b^	√	√	–	–	Tumor progression- 3rd relapse No MAT and ASCT due to pulmonary hypertension; receiving 3rd-line chemotherapy (including lorlatinib) Died of disease: 7 yrs 8 m of age
10C	F	8 m	4	Retroperitoneum	Liver, bone	*MYCN* amplified del(1p) unknown	NB2004	MR	√	–	–	–	–	Died of disease: tumor progression 1 yr 7 m of age
11C	M	2 yrs 7 m	4	Left suprarenal gland	BM	*MYCN* amplified del(1p) unknown	NB2004	VGPR	√	–	–	BuMel	CR	Died of sepsis in post-MAT period 3 yrs 1 m of age
12C	F	2 yrs 6 m	4	Retroperitoneum	BM, bone	*MYCN* amplified del(1p) unknown	NB2004	MR	√	–	–	BuMel	VGPR	Died of disease: tumor progression 4 yrs 2 m of age

^a^ All patients had tumors that were poorly differentiated or not otherwise specified, based on the Shimada classification. ^b^ Including lymphadenectomy. ASCT, autologous stem cell transplant (peripheral blood); BM, bone marrow; BuMel, high-dose busulfan and melphalan; C, control; CEM, carboplatin, etoposide, melphalan; CR, complete response; del, deletion; F, female; FU, follow up; ^131^I-MIBG, iodine-131-metaiodobenzylguanidine; INSS, International Neuroblastoma Staging System; M, male; m, months; MAT, myeloablative therapy; MR, mixed response; NB2004, 2 cycles of N8 (topotecan, cyclophosphamide, etoposide) and 6 cycles of alternating N5 (cisplatin, etoposide, vindesine) and N6 (vincristine, dacarbacine, ifosfamide, doxorubicin); PD, progressive disease; PR, partial response; pt, patient; RT, radiotherapy; VGPR, very good partial response; yr(s), years (of age).

**Table 3:** Disease characteristics, treatment and outcomes for 11 patients with high-risk neuroblastoma who received immunotherapy with dinutuximab beta.

Pt	Sex	Age at Diagnosis (*Relapse*)	INSS Stage [18]	Primary Tumor (Pathophysiologic Findings)	Metastases	Mutations *MYCN* del(1p)		Treatment and Status of Disease							Outcome (Last FU)
							Induction Chemotherapy	Status AfterInduction Chemotherapy	Surgery	RT	^131^I-MIBG	MAT	Status after MAT and ASCT	DB ^a^	
1	F	2 yrs 2 m	4	Right suprarenal gland (NOS)	Bone, BM	*MYCN* amplified del(1p) positive	NB2004	VGPR	√	–	–	BuMel	VGPR	√ + iso	Tumor classified as ganglioneuroblastoma following chemotherapy Died of disease: leptomeningeal dissemination; 4 yrs of age
2	M	4 yrs 5 m	4	Left suprarenal gland (NOS)	Bone, BM	*MYCN* amplified del(1p) unknown	NB2004	VGPR	√ ^b^	–	–	BuMel	CR	√ + iso	Complete remission duration: 4 yrs 10 m (last FU Oct 2021; 9 yrs 10 m of age)
3	F	10 m	4	Retroperitoneum (poorly differentiated)	Bone, great vessels involved	*MYCN* not amplified del(1p) negative	NB2004	VGPR	–	–	√	1. CEM2. BuMel	CR	√ + iso	Complete remission duration: 5 yrs 8 m (last FU Dec 2021; 7 yrs 1 m of age)
4	F	1 yr 1 m *Relapse*: 10 yrs 5 m	4	Right suprarenal gland (poorly differentiated)	Bone, great vessels dislocated, retrocrural lymph nodes	*MYCN* amplified del(1p) unknown	NB2004	VGPR 2nd VGPR	√ ^b^	–	√	CEM *Relapse*: 1. CEM 2. BuMel	VGPR 2nd VGPR	√ + IL-2	Complete remission duration: 5 yrs 6 m (last FU Jan 2022; 17 yrs 1 m of age)
5	F	2 yrs 8 m	4	Left suprarenal gland (poorly differentiated)	Bone	*MYCN* not amplified del(1p) unknown	NB2004	VGPR	√ ^b^	–	√	1. CEM2. BuMel	CR	√ + IL-2	Complete remission ^c^ duration: 6 yrs 1 m (last FU Oct 2021; 8 yrs of age)
6	M	2 yrs 2 m	4	Intra-abdominal (poorly differentiated)	Lungs, liver, bone	*MYCN* not amplified del(1p) negative	NB2004	VGPR	√ ^b^	–	√	BuMel	VGPR	√ (×1)	Died of disease: Tumor progression after one cycle of DB; 4 yrs of age
7	F	5 m *Relapse:* 1 yr 3 m	4	Intra-abdominal (NOS)	BM, liver	*MYCN* not amplifieddel(1p) unknown	NB2004 (intermediate risk) *Relapse:* N8	VGPR	–	–	√	CEM	VGPR	√ + IL-2	Complete remission duration: 6 yrs 2 m (last FU Oct 2021; 8 yrs 3 m of age)
8	M	2 yrs 10 m	4	Left suprarenal gland (NOS)	Bone, BM	*MYCN* not amplified del(1p) negative	NB2004	VGPR	√	–	√	BuMel	CR	√ + iso ^d^	Complete remission duration: 4 yrs 5 m (last FU Jan 2022; 7 yrs 5 m of age)
9	F	3 yrs 6 m	4	Right suprarenal gland (poorly differentiated)	Bone, BM, pancreas	*MYCN* not amplified del(1p) unknown	Rapid COJEC	VGPR	√ ^b^	√	–	BuMel	CR	√	Complete remission duration: 2 yrs 5 m (last FU April 2022; 6 yrs 10 m of age)
10	M	1 yr 10 m	4	Left suprarenal gland (NOS)	Bone, BM	*MYCN* amplified del(1p) unknown	Rapid COJEC & TVD After 2nd surgery: irinotecan- temozolomide	VGPR	√ ^e^	√	–	BuMel	CR	√ (×4)	Stable disease duration: 1 yr 1 m (last FU April 2022; 5 yrs 6 m of age) DB stopped after 4 cycles due to facial paresis
11	F	10 m	4	Mediastinum (poorly differentiated; neuroblastoma/ganglioneuroblastoma)	–	*MYCN* amplified del(1p) unknown	Rapid COJEC	VGPR	√	√	–	BuMel ^f^	CR	√	Complete remission duration: 1 yr 3m (last FU Feb 2022; 2 yrs 7 m of age)

^a^ Number of cycles, if less than the 5 cycles of dinutuximab beta recommended. ^b^ Including lymphadenectomy. ^c^ Patient developed AML and underwent BMT following immunotherapy, but is now in remission. ^d^ Patients received 5 cycles of dinutuximab beta, but only 2 cycles of isotretinoin (due to diarrhea). ^e^ Patient underwent two surgeries (macroscopic tumor resection followed later by an extirpation of residual tumor). ^f^ Plus 10 days of defibrotide to prevent veno-occlusive disease. AML, acute myeloid leukemia; ASCT, autologous stem cell transplant (peripheral blood); BM, bone marrow; BMT, bone marrow transplantation; CEM, carboplatin, etoposide, melphalan; CR, complete remission; DB, dinutuximab beta; del, deletion; F, female; FU, follow up; ^131^I-MIBG, iodine-131-metaiodobenzylguanidine; IL-2, interleukin 2; INSS, International Neuroblastoma Staging System; iso, isotretinoin; M, male; m, months; MAT, myeloablative therapy; N8, topotecan, cyclophosphamide, and etoposide; NB2004, 2 cycles of N8 and 6 cycles of alternating N5 (cisplatin, etoposide, vindesine) and N6 (vincristine, dacarbazine, ifosfamide, doxorubicin); NOS, not otherwise specified; rapid COJEC, time-intensive cisplatin (C), vincristine (O), carboplatin (J), etoposide (E), and cyclophosphamide (C); RT, radiotherapy; TVD, topotecan (T), vincristine (V), and doxorubicin (D); VGPR, very good partial response; yr(s), years of age.

**Table 4:** Grade 3/4 adverse events occurring during each cycle of dinutuximab beta therapy in patients treated with or without IL-2.

Adverse Events, n	Cycle 1		Cycle 2		Cycle 3		Cycle 4		Cycle 5	
	DB	DB + IL-2	DB	DB + IL-2	DB	DB + IL-2	DB	DB + IL-2	DB	DB + IL-2
Pyrexia/hyperpyrexia	2	4	1	0	0	0	0	0	0	0
Fluid retention	2	2	1	0	0	0	0	0	0	0
Hypotension	1	3	1	0	0	0	0	0	0	0
Hypoxia	2	1	1	0	0	0	0	0	0	0
Diarrhea	1	2	0	0	0	0	0	0	0	0
Pain	2	0	0	0	0	0	0	0	0	0
Hypertension	1	0	0	0	0	0	0	0	0	0
Renal impairment	0	0	0	0	1	0	0	0	0	0
Facial paresis	0	0	0	0	0	0	1	0	0	0
Tachycardia	1	0	0	0	0	0	0	0	0	0
Blurred vision	1	0	0	0	0	0	0	0	0	0

DB, dinutuximab beta; IL-2, interleukin-2.

## Data Availability

The data presented in this study are available on reasonable request from the corresponding author.

## References

[1] Swift C.C., Eklund M.J., Kraveka J.M., Alazraki A.L. (2018). Updates in Diagnosis, Management, and Treatment of Neuroblastoma. Radiographics.

[2] Georgakis M.K., Dessypris N., Baka M., Moschovi M., Papadakis V., Polychronopoulou S., Kourti M., Hatzipantelis E., Stiakaki E., Dana H. (2018). Neuroblastoma among children in Southern and Eastern European cancer registries: Variations in incidence and temporal trends compared to US. Int. J. Cancer.

[3] Spix C., Pastore G., Sankila R., Stiller C.A., Steliarova-Foucher E. (2006). Neuroblastoma incidence and survival in European children (1978-1997): Report from the Automated Childhood Cancer Information System project. Eur. J. Cancer.

[4] Liang W.H., Federico S.M., London W.B., Naranjo A., Irwin M.S., Volchenboum S.L., Cohn S.L. (2020). Tailoring Therapy for Children With Neuroblastoma on the Basis of Risk Group Classification: Past, Present, and Future. JCO Clin. Cancer Inform..

[5] Zafar A., Wang W., Liu G., Wang X., Xian W., McKeon F., Foster J., Zhou J., Zhang R. (2021). Molecular targeting therapies for neuroblastoma: Progress and challenges. Med. Res. Rev..

[6] Chung C., Boterberg T., Lucas J., Panoff J., Valteau-Couanet D., Hero B., Bagatell R., Hill-Kayser C.E. (2021). Neuroblastoma. Pediatr. Blood Cancer.

[7] Ladenstein R., Potschger U., Pearson A.D.J., Brock P., Luksch R., Castel V., Yaniv I., Papadakis V., Laureys G., Malis J. (2017). Busulfan and melphalan versus carboplatin, etoposide, and melphalan as high-dose chemotherapy for high-risk neuroblastoma (HR-NBL1/SIOPEN): An international, randomised, multi-arm, open-label, phase 3 trial. Lancet Oncol..

[8] Ladenstein R., Pötschger U., Valteau-Couanet D., Luksch R., Castel V., Yaniv I., Laureys G., Brock P., Michon J.M., Owens C. (2018). Interleukin 2 with anti-GD2 antibody ch14.18/CHO (dinutuximab beta) in patients with high-risk neuroblastoma (HR-NBL1/SIOPEN): A multicentre, randomised, phase 3 trial. Lancet Oncol..

[9] Ladenstein R., Pötschger U., Valteau-Couanet D., Luksch R., Castel V., Ash S., Laureys G., Brock P., Michon J.M., Owens C. (2020). Investigation of the Role of Dinutuximab Beta-Based Immunotherapy in the SIOPEN High-Risk Neuroblastoma 1 Trial (HR-NBL1). Cancers.

[10] Yu A.L., Gilman A.L., Ozkaynak M.F., London W.B., Kreissman S.G., Chen H.X., Smith M., Anderson B., Villablanca J.G., Matthay K.K. (2010). Anti-GD2 antibody with GM-CSF, interleukin-2, and isotretinoin for neuroblastoma. N. Engl. J. Med..

[11] Yu A.L., Gilman A.L., Ozkaynak M.F., Naranjo A., Diccianni M.B., Gan J., Hank J.A., Batova A., London W.B., Tenney S.C. (2021). Long-Term Follow-up of a Phase III Study of ch14.18 (Dinutuximab) + Cytokine Immunotherapy in Children with High-Risk Neuroblastoma: COG Study ANBL0032. Clin. Cancer Res..

[12] Ladenstein R.L., Poetschger U., Valteau-Couanet D., Gray J., Luksch R., Balwierz W., Castel V., Ash S., Popovic M.B., Laureys G. (2019). Randomization of dose-reduced subcutaneous interleukin-2 (scIL2) in maintenance immunotherapy (IT) with anti-GD2 antibody dinutuximab beta (DB) long-term infusion (LTI) in front–line high-risk neuroblastoma patients: Early results from the HR-NBL1/SIOPEN trial. J. Clin. Oncol..

[13] Lode H.N., Valteau-Couanet D., Gray J., Luksch R., Wieczorek A., Castel V., Ash S., Laureys G., Papadakis V., Owens C. (2019). Randomized use of anti-GD2 antibody dinutuximab beta (DB) long-term infusion with and without subcutaneous interleukin-2 (scIL-2) in high-risk neuroblastoma patients with relapsed and refractory disease: Results from the SIOPEN LTI-trial. J. Clin. Oncol..

[14] EUSA Pharma Qarziba (Dinutuximab Beta) Summary of Product Characteristics. https://www.ema.europa.eu/en/documents/product-information/qarziba-epar-product-information_en-0.pdf.

[15] High-Risk Neuroblastoma: Standard Clinical Practice Recommendations. https://siope.eu/media/documents/escp-high-risk-neuroblastoma-standard-clinical-practice-recommendations.pdf.

[16] Simon T. S1-Leitlinie 025-008 Neuroblastom. https://www.awmf.org/uploads/tx_szleitlinien/025-008l_S1_Neuroblastom_2019-07_01.pdf.

[17] Blonde L., Khunti K., Harris S.B., Meizinger C., Skolnik N.S. (2018). Interpretation and Impact of Real-World Clinical Data for the Practicing Clinician. Adv. Ther..

[18] Brodeur G.M., Pritchard J., Berthold F., Carlsen N.L., Castel V., Castelberry R.P., De Bernardi B., Evans A.E., Favrot M., Hedborg F. (1993). Revisions of the international criteria for neuroblastoma diagnosis, staging, and response to treatment. J. Clin. Oncol..

[19] Berthold F., Faldum A., Ernst A., Boos J., Dilloo D., Eggert A., Fischer M., Fruhwald M., Henze G., Klingebiel T. (2020). Extended induction chemotherapy does not improve the outcome for high-risk neuroblastoma patients: Results of the randomized open-label GPOH trial NB2004-HR. Ann. Oncol..

[20] Simon T., Hero B., Schulte J.H., Deubzer H., Hundsdoerfer P., von Schweinitz D., Fuchs J., Schmidt M., Prasad V., Krug B. (2017). 2017 GPOH Guidelines for Diagnosis and Treatment of Patients with Neuroblastic Tumors. Klin. Padiatr..

[21] Barone G., Barry A., Bautista F., Brichard B., Defachelles A.S., Herd F., Manzitti C., Reinhardt D., Rubio P.M., Wieczorek A. (2021). Managing Adverse Events Associated with Dinutuximab Beta Treatment in Patients with High-Risk Neuroblastoma: Practical Guidance. Paediatr. Drugs.

[22] Achbergerová M., Hederová S., Mikesková M., Husáková K., Hrašková A., Kolenová A. (2020). Implementation of immunotherapy into the treatment of neuroblastoma–single center experience with the administration of dinutuximab and management of its adverse effects. Klin. Onkol..

[23] Achbergerová M., Hederová S., Hrašková A., Kolenová A. (2022). Dinutuximab beta in the treatment of high-risk neuroblastoma: A follow-up of a case series in Bratislava. Medicine.

